# Elevated microsatellite alterations at selected tetranucleotides in early‐stage colorectal cancers with and without high‐frequency microsatellite instability: same, same but different?

**DOI:** 10.1002/cam4.709

**Published:** 2016-04-06

**Authors:** Martin M. Watson, Dordi Lea, Emma Rewcastle, Hanne R. Hagland, Kjetil Søreide

**Affiliations:** ^1^ Department of Gastrointestinal SurgeryStavanger University HospitalStavangerNorway; ^2^ Gastrointestinal Translational Research UnitMolecular Laboratory, HillevågStavanger University HospitalStavangerNorway; ^3^ Department of PathologyStavanger University HospitalStavangerNorway; ^4^ Centre for Organelle Research (CORE)University of StavangerStavangerNorway; ^5^ Department of Clinical MedicineUniversity of BergenBergenNorway

**Keywords:** Colorectal cancer, elevated microsatellite alterations, microsatellite instability, node status, survival

## Abstract

Microsatellite instability (MSI) is associated with better prognosis in colorectal cancer (CRC). Elevated microsatellite alterations at selected tetranucleotides (EMAST) is a less‐understood form of MSI. Here, we aim to investigate the role of EMAST in CRC±MSI related to clinical and tumor‐specific characteristics. A consecutive, population‐based series of stage I–III colorectal cancers were investigated for MSI and EMAST using PCR primers for 10 microsatellite markers. Of 151 patients included, 33 (21.8%) had MSI and 35 (23.2%) were EMAST+, with an overlap of 77% for positivity, (odds ratio [OR] 61; *P* < 0.001), and 95% for both markers being negative. EMAST was more prevalent in colon versus rectum (86% vs. 14%, *P* = 0.004). EMAST+ cancers were significantly more frequent in proximal colon (77 vs. 23%, *P* = 0.004), had advanced t‐stage (T3–4 vs. T1–2 in 94% vs. 6%, respectively; *P* = 0.008), were larger (≥5 cm vs. <5 cm in 63% and 37%, respectively; *P* = 0.022) and had poorly differentiated tumor grade (71 vs. 29%, *P* < 0.01). Furthermore, EMAST+ tumors had a higher median number of harvested lymph nodes than EMAST− (11 vs. 9 nodes; *P* = 0.03). No significant association was found between EMAST status and age, gender, presence of distant metastases or metastatic lymph nodes, and overall survival. A nonsignificant difference toward worse survival in node‐negative colon cancers needs confirmation in larger cohorts. EMAST+ cancers overlap and share features with MSI+ in CRC. Overall, survival was not influenced by the presence of EMAST, but may be of importance in subgroups such as node‐negative disease of the colon.

## Introduction

Colorectal cancer (CRC) remains a formidable global health burden and represents one of the most frequent tumors in both genders 1. Prognosis and treatment decisions are still largely based on the TNM system, but despite revisions to improve its predictive and prognostic value, this system is still under debate 2, 3. Among the strongest prognostic factors is lymph node status, with node‐positivity usually indicating a less favorable prognosis and a need for adjuvant chemotherapy after surgery. However, even the role of lymph nodes has been debated 4, as this is a fairly rough quality indicator and fails to avoid under‐ and overtreatment. The growing evidence for the role of genetic variability in cancer behavior and disease outcome has therefore called for a stratified approach to cancer care based on specific molecular traits.

The last decades have shed light on several important molecular mechanisms of CRC, allowing for useful clinical subtyping and making CRC a useful model for the understanding of cancer initiation and progression 5. Microsatellite instability (MSI) is one such important feature and has been associated with better prognosis and tumor‐specific characteristics 6. First described in the hereditary proportion of CRCs and associated with the Lynch syndrome, MSI also occurs in about 15% of sporadic CRCs.

MSI represents a pathway of carcinogenesis that runs parallel to that of chromosomal instability and is of acknowledged prognostic, predictive, and potentially therapeutic relevance 6. Instability at mono‐ and dinucleotide microsatellites is today included in the clinical and biological definition of MSI, for example, by the Bethesda criteria for MSI testing and definitions 7. However, in a rapidly increasing number of studies, instability at tetranucleotides has been described and considered as a particular subtype of MSI over a wide range of tumor types, from those originating in the aerodigestive organs to the gastrointestinal tract 8. This newly described form of microsatellite instability was named “elevated microsatellite alterations at selected tetranucleotides” (EMAST).

In CRC, several recent findings have suggested potential molecular mechanisms underlying EMAST 9, 10, 11, 12, 13. However, the clinicopathological relevance and difference with “canonical” MSI in CRC is still poorly investigated. Thus, we aim to investigate the role of EMAST in relation to clinical‐ and tumor‐specific data, including MSI status, and analyze the effect on survival.

## Patients and Methods

### Study cohort

The study cohort represents consecutive patients with non‐metastatic colorectal cancer (stage I–III) who were <75 years of age at diagnosis and who entered into an in‐hospital, surgeon‐led systematic surveillance program per national standards at the time^14^, and as previously described at the time 15, 16, 17. All included patients presented between 1996 and 1999 and underwent curative surgery for colorectal cancer at the Department of Gastrointestinal Surgery, Stavanger University Hospital, Norway. Clinicopathological information was recorded, and follow‐up was updated as of July 23rd, 2011, thus providing up to 15 years follow‐up after surgery 16.

Notably, as this cohort represents patients who were eligible for a systematic surveillance program at the time of surgery, patient >75 years and stage III (pN+) not fit for adjuvant chemotherapy were excluded 15, 16. Patients with stage III disease and who were otherwise fit were offered adjuvant chemotherapy according to national guidelines at the time, typically consisting of 5‐fluorouracil and leucovorin (5‐FU/LV) 18. Thus, elderly patients and those deemed unfit for adjuvant chemotherapy or, in the case of distant recurrence, deemed not fit for a second surgery were not included in this cohort.

From the above‐described initial cohort (*n* = 196), there were 151 specimens (98 from colon and 53 from rectum) with available tissue for DNA extraction from formalin‐fixed, paraffin‐embedded (FFPE) tumor and tumor‐free resection margin tissues for this study.

### Ethics

The study was approved as a quality assurance project (REK#2010/3414) by the Regional Ethics Committee of the Health Trust of Western Norway.

### Gross and histopathological assessment

All tumors were assessed for gross and histomorphological characteristics, and staged according to TNM‐classifications per routine at the time. For the current analyses, a pathologist reviewed the slides to ensure appropriate selection of tumor tissue and blocks with appropriate high tumor content (>50% viable tumor tissue) per block used for DNA extraction.

### DNA extraction and fragment analysis

Following inspection by an experienced pathologist, four consecutive tumor and tumor‐free 10 *μ*m sections were cut from FFPE blocks for DNA extraction, using the Tissue DNA E.Z.N.A. kit (Omega BioTech^®^, Norcross, GA, USA) according to manufacturer's instructions. DNA extracted from tumor tissue and their corresponding normal tissue (from surgical resection margins) was then PCR‐amplified with five tetranucleotide microsatellites primer pairs (EMAST: D20S85, D20S82, D9S242, D8S321, MYCL1, 5` fluorescently labeled) and five mono‐ and dinucleotide microsatellite primer pairs (MSI: NR‐27, NR‐21, NR‐24, BAT‐25, BAT‐26, 5` fluorescently labeled). PCR conditions were as follows: initial denaturation step of 5′ at 95°C, followed by 37 cycles of denaturation (30″ at 95°C), annealing (90″ at 55°C), and extension (30″ at 72°C), and concluded by a final elongation step (30′ at 60°C). The primers sequences, expected amplicon sizes and fluorescent dyes are provided in Table 1.

**Table 1:** Name, size, fluorescent label, and primer sequences of the microsatellite markers investigated

Marker	Amplicon size (bp)	Label	Forward primer	Reverse primer
*EMAST primers*				
MYCL1	181	6‐FAM	TGGCGAGACTCCATCAAAG	CCTTTTAAGCTGCAACAATTTC
D20S85	146	NED	GAGTATCCAGAGAGCTATTA	ATTACAGTGTGAGACCCTG
D8S321	237	VIC	GATGAAAGAATGATAGATTACAG	ATCTTCTCATGCCATATCTGC
D20S82	249	6‐FAM	GCCTTGATCACACCACTACA	GTGGTCACTAAAGTTTCTGCT
D9S242	178	PET	GTGAGAGTTCCTTCTGGC	ACTCCAGTACAAGACTCTG
*MSI primers*				
NR‐27	89	VIC	AACCATGCTTGCAAACCACT	CGATAATACTAGCAATGACC
NR‐21	110	6‐FAM	GAGTCGCTGGCACAGTTCTA	CTGGTCACTCGCGTTTACAA
NR‐24	128	PET	GCTGAATTTTACCTCCTGAC	ATTGTGCCATTGCATTCCAA
BAT‐25	152	VIC	TACCAGGTGGCAAAGGGCA	TCTGCATTTTAACTATGGCTC
BAT‐26	182	NED	CTGCGGTAATCAAGTTTTTAG	AACCATTCAACATTTTTAACCC

EMAST, Elevated microsatellite alterations at selected tetranucleotides; MSI, Microsatellite instability.

The PCR products were analyzed for fragment lengths on a 3130xl GeneticAnalyzer (Applied Biosystems, Foster City, CA, USA), with GeneMapper v3.7 software (Applied Biosystems, Foster City, CA, USA). Tumor samples were compared with their corresponding normal samples. Those showing any number of extra peaks at ±4*n* (n ≠ 0) (tetranucleotides markers, EMAST), and/or ±1*n* or 2*n* (n ≠ 0) (mono‐ and dinucleotide markers, respectively, MSI) were scored as unstable for that marker.

### Definition of EMAST and MSI

To detect EMAST either direct sequencing or fragment analysis are generally used, with most laboratories adopting a panel of five tetranucleotide polymorphic markers (at least two unstable markers to score EMAST positivity). In CRC, up to seven microsatellite markers have been reportedly used, with EMAST considered present (EMAST+) when at least one marker was found unstable. In this study, we adopt the most used definitions of at least two out of five tetranucleotide markers unstable to confirm EMAST.

Samples showing instability in at least two out of five markers (40%) were recorded as EMAST‐positive and/or microsatellite instability‐high (MSI‐H), while instability of one out of five markers was scored as EMAST‐negative and/or microsatellite instability‐low (MSI‐L). If no unstable markers were found, the specimens were considered as microsatellite‐stable (MSS). MSI analysis was done as previously described 17, 19. Two investigators completed the scoring process independently, blinded to each other's results. Discordance among investigators' scoring was addressed by rerunning the samples by PCR followed by rescoring.

### Statistical analysis

All statistical analyses were performed on IBM^®^ SPSS^®^ Statistics for Mac and Windows, version 23 (Armonk, NY, USA). Continuous variables were tested for normality by the Shapiro–Wilks test and for comparison by Mann–Whitney U test. Relationships between categorical variables were investigated via Fischer's exact and Chi‐square tests, as appropriate. Overall and recurrence‐free survival was assessed by Kaplan–Meier analysis using the log rank test. All tests are two‐tailed and statistical significance was set at *P *<* *0.050.

## Results

**Figure 1:**
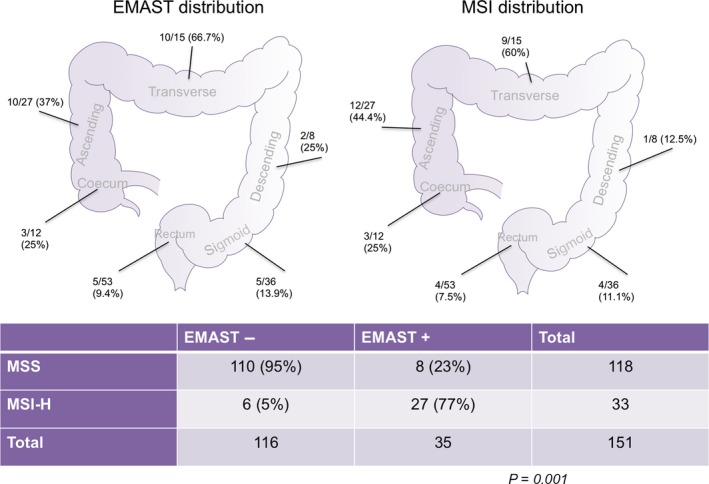
Elevated microsatellite alterations at selected tetranucleotides (EMAST) and microsatellite instability (MSI) cancer distribution in colon and rectum. EMAST denotes elevated microsatellite alterations at selected tetranucleotides; MSI denotes microsatellite instability.

Of the 151 patients included, the age and gender distribution together with other clinicopathological characteristics are presented in Table**** 2. The frequencies of MSI‐H and EMAST were of 33 and 35 (22 and 23%) out of 151 patients included, respectively (Table 2). Seventy‐seven percent of EMAST cases (27/35) were also MSI‐H for an odds ratio (OR) of 61.9, (95% CI: 19.8–193.3; *P* < 0.001). The distribution of MSI‐ and EMAST‐positive tumors across the different sections of the large intestine are presented in Figure**** 1. Dual positive cases (both EMAST+ and MSI+; *n* = 27), were predominantly located in the colon (*n* = 25; 92.6%) compared to rectum (*n* = 2; 7.8%). The ascending and transverse colon had the highest number of dual positive cases, for seven and eight each (25.9% and 29.6%, respectively).

**Table 2:** Characteristics of patient and tumors according to EMAST status

		*n* (%)		*P*	*n* (%)		*P*
*n* = 151		EMAST−	EMAST+		MSS	MSI	
Age (years)	<65	58 (73.4)	21 (26.6)	*0.299*	57 (72.2)	22 (27.8)	*0.062*
	≥65	58 (80.6)	14 (19.4)		61 (84.7)	11 (15.3)	
Gender	M	73 (79.3)	19 (20.7)	*0.358*	75 (81.5)	17(18.5)	*0.210*
	F	43 (72.9)	16 (27.1)		43 (72.9)	16 (27.1)	
Tumor location	Colon	68 (69.4)	30 (30.6)	***0.003***	69 (70.4)	29 (29.6)	***0.002***
	Rectum	48 (90.6)	5 (9.4)		49 (92.5)	4 (7.5)	
Tumor stage	T_1–2_	31 (93.9)	2 (6.1)	***0.008***	31 (93.9)	2 (6.1)	***0.013***
	T_3**–**4_	85 (72.0)	33 (28.0)		87 (73.7)	31 (26.3)	
Tumor grade	Poor/mucinous	10 (50.0)	10 (50.0)	***0.002***	8 (40.0)	12 (60.0)	***<0.001***
	Moderate/well	106 (80.9)	25 (19.1)		110 (84.0)	21 (16.0)	
Tumor size^a^	≥5 cm	44 (68.8)	20 (31.2)	***0.022***	43 (67.2)	21 (32.8)	***<0.001***
	<5 cm	67 (84.8)	12 (15.2)		72 (91.1)	7 (8.9)	

EMAST, Elevated microsatellite alterations at selected tetranucleotides; MSI, microsatellite instability; MSS, microsatellite stable. Values highlighted in bold indicate significance level of *p* < 0.05.

a Size missing in eight samples (5.3%)

Of the 53 rectal and 98 colon tumors, five (9.4%) and 30 (30.6%) were positive for EMAST, respectively (cumulative: 86% colon, 14% rectum, *P* = 0.004). EMAST+ tumors had a higher prevalence in proximal versus distal colon (77% vs. 23%, *P* = 0.004) and were also associated with advanced t‐stage in both EMAST (OR 6.0, 95% CI: 1.4–26.6; *P* = 0.008) and MSI cancers (OR 5.5, 95% CI: 1.3–24.5; *P* = 0.013), respectively.

**Figure 2:**
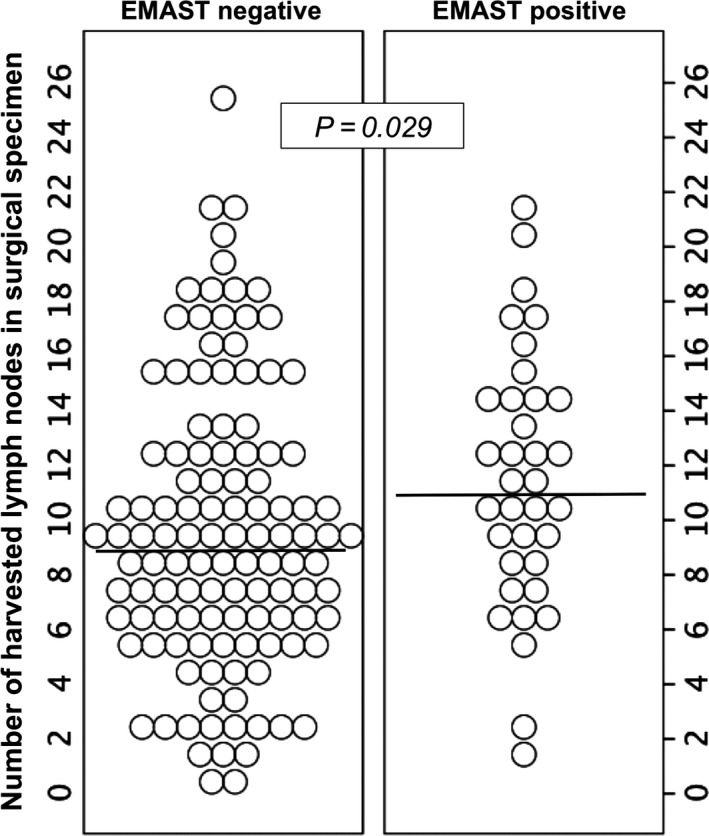
Number of lymph nodes found in the resected specimen, according to elevated microsatellite alterations at selected tetranucleotides (EMAST) status. *P*‐value for difference in median number between groups.

EMAST+ tumors had a higher median number of harvested lymph nodes than EMAST– (11 vs. 9 nodes; *P* = 0.029; Fig.**** 2), but no difference in the number of lymph nodes positive for tumor cells infiltration was found.

**Figure 3:**
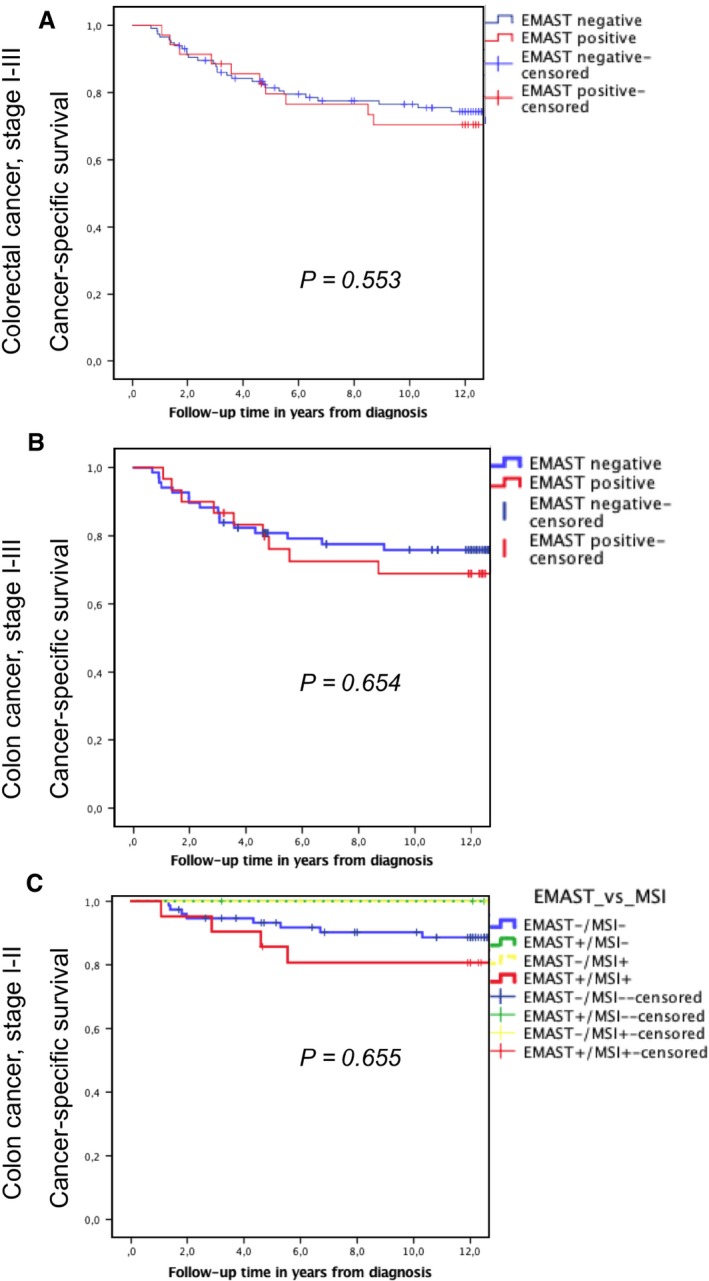
Cancer‐specific survival according to elevated microsatellite alterations at selected tetranucleotides (EMAST) status, stages, and location. (A) overall cancer‐specific survival for all colorectal stage I–III cancers, with no significant difference, yet a somewhat poorer outcome in EMAST + cancers. (B) cancer‐specific survival for colon cancers only, again with nonsignificant poorer survival in EMAST+ patients. (C) outcome for node‐negative (stage I–II) colon cancers, split into patients with microsatellite stable (MSS; blue line), microsatellite instability (MSI)‐/EMAST+ (green), MSI+/EMAST− (yellow), and the dual positive cancers of EMAST+ and MSI+ (red), with poorest outcome.

A total of 38 (25.2%) patients developed metastases and died from CRC in this cohort. Neither EMAST nor MSI predicted risk of development of distant metastases, nor was EMAST predictive for disease‐specific and for overall long‐term survival in this cohort (Fig. 3A). However, on subanalyses of colon cancers only, as these harbor a higher frequency of EMAST‐positive cases in comparison with rectal cancers, a nonsignificant difference in long‐term cancer‐specific survival was noted, particularly for the node‐negative (stage I–II) patients (Fig. 3B). Furthermore, these depicted an apparent worse outcome for EMAST+ (Fig.**** 3C) compared to microsatellite‐stable cancers, and those with either one form of microsatellite instability only.

## Discussion

In this cohort study, we found EMAST+ CRC to largely overlap with features associated with MSI+ cancers, including a predominant location in the colon, association with low‐grade histology, larger tumor size, and advanced depth of growth (T3‐4). Despite a higher number of lymph nodes sampled for EMAST+ cancers, there was no difference in the number of malignant nodes (neither in actual numbers nor in the rate of pN+ cases) and no statistically significant effect on survival could be found. The nonsignificant yet apparent difference in survival curves between groups depicted in Figure 3 with a trend toward reduced survival in EMAST+ cancers, specifically for node‐negative colon cancers needs verification in larger cohorts.

Several findings need comment in this study. We found a significantly lower prevalence of EMAST in our sample (23%) when compared with the majority of the studies available in the literature, reporting EMAST frequencies in CRC ranging between 33% and 64.8% 9, 12, 13, 20, 21, 22. As previously reviewed 8, such variation could be due to the type and number of markers and the thresholds used in EMAST analyses, with the highest (60% and over) frequencies reported by groups using a less stringent approach to define EMAST 22. The degree of overlap between MSI cases and EMAST (77% in this study), is in line with studies that report between 67% and 100% overlap, and thus, the selected cohort should be within the range of variation as reported elsewhere in the literature. The fact that most, and in some cases all, the MSI‐H tumors are also EMAST+, and that the latter tumors are generally more prevalent, could suggest a cause–consequence relationship. It is also interesting to see the prognostic features shared by MSI and EMAST‐positive cancers (Table 2), including larger size, poor differentiation, and depth of growth. While these features would normally be associated with a worse prognosis, some emerging data suggest that MSI and possibly EMAST cancers may be associated with specific immune reactions and T‐lymphocyte infiltrations associated with a more favorable outcome 10.

Neither EMAST nor MSI was found to significantly correlate with survival (neither overall nor recurrence‐free). Of the four studies investigating survival, specifically in EMAST+ and EMAST− tumors currently available in the scientific literature, two found no significant difference in overall survival 9, 12. However, one study of metastatic CRC observed that MSI‐H tumors that also displayed EMAST+ had significantly worse overall survival, compared to non‐EMAST cancers with MSI‐H 13. This is in line with the nonsignificant findings in this study, albeit in early‐stage colon cancers. A further study found a significantly reduced recurrence‐free survival (RFS) in EMAST+ cancers, when compared to MSI‐H, but the degree of overlapping EMAST/MSI was not disclosed 23. In both studies the MSI‐H/EMAST‐ group was composed of a limited number of individuals.

As demonstrated in the current cohort, both MSI and EMAST produce larger tumors. If this is because of EMAST/MSI tumors developing more quickly due to a much higher rate of mutation that a defective MMR system confers to the nest of cancer‐initiating cells remains speculative, but warrants investigation. Conversely, EMAST/MSI may be genetic events that occur as a side effect of other drivers of carcinogenesis, or merely reflects a high turnover and induction of genetic errors during rapid growth of the tumor cells. Some studies look into mechanisms of EMAST 11, 22, 24, 25, and point to a role of hypoxia, oxidative stress, and DNA repair mechanisms 26. However, overall data are scarce and further understanding is thus needed.

Some limitations should be considered when comparing our study cohort with other patient series. First, we included only patients who were <75 years and who entered a systematic surveillance program after surgery 15, and excluded elderly patients or those with comorbidities who were unfit for adjuvant chemotherapy or unlikely to tolerate metastatic surgery. Thus, we have introduced a clinical bias towards younger, fitter patients with stage I–III colorectal cancers in this series. This should be taken into account when interpreting our findings, as the results could thus not apply to a more general patient cohort with higher age and that frequently included stage IV cancers. The latter may also be the reason for a nonsignificant trend in the analyses, as others have found EMAST status of importance in stage IV and metastatic disease 13, 23, 27. For example, Venderbosch et al.^13^ found a statistically significant difference in survival between patients with and without EMAST. Notably, the included patients all came from clinical phase III trials (the Dutch CAIRO and CAIRO2 studies) of metastatic CRC, and thus all patients in the cohort had an unfavorable outcome 13. Indeed, EMAST may be an accumulated effect of worse biology, higher mutational load, and thus play a more prominent role in biology in late stage (e.g., metastatic disease) compared with early (stage I–III) disease. Evidence that EMAST may act as a potential biological modulator among the different types of molecular classes (e.g., microsatellite instability, epigenetics, and chromosomal instability) involved in CRC have been proposed 28, and is further suggested in a combined series of metastatic disease in CRC 27. Thus, EMAST may be more specific for tumor biology and disease outcome in late‐stage groups 29, such as colorectal liver metastasis, but further studies need to corroborate these findings. Notably, several aspects in clinical practice, including a higher frequency of metastatic surgery and extending adjuvant chemotherapy to elderly patients have occurred since the late 1990s, so clinical differences in practice may have introduced selection and outcome bias in this cohort compared with more recent cohorts. However, long‐term follow‐up would not be possible with more recent cohorts, so the true eventual outcome of the patients (e.g., death from disease or other cause; still alive with no evidence of disease etc.) is likely to have been captured accurately in this series. The small cohort prevents from robust subgroup analysis and these should therefore be interpreted with caution. An apparent prognostic role in stage I–II CRC warrants further investigation. Finally, how EMAST should be determined lacks firm definition in the current literature, possibly explaining why our results deviate from others based on the choice of defined markers and numbers used for positivity. This methodological issue must be solved through further clarification of biological mechanisms and ability for robust and valid tests of selected markers or panels of markers.

While the clinical role of EMAST in CRC is still being investigated, the biological implications of recent investigations may yield findings of new mechanisms that have a clinical relevance in selected patients at both extremes of presentation, either as early cancers or as metastatic disease. Thus, further investigation into the biological mechanisms and their potential clinical implications should be pursued. Whether EMAST is an epiphenomenon or a specific genetic trait warrants further investigation.

## Conflict of Interest

None declared.
